# RIBOFLAVIN deficiency in rheumatic Diseases: A call for clinical trials

**DOI:** 10.1515/rir-2026-0024

**Published:** 2026-07-13

**Authors:** Jozélio Freire de Carvalho, Rosangela Passos de Jesus

**Affiliations:** Núcleo de Pesquisa em Nutrição e Doenças Crônicas (NUPEN-DC), School of Nutrition, Federal University of Bahia, Salvador, BA, Brazil

Dear Editor,

Riboflavin (vitamin B^2^) is a water-soluble B-vitamin that functions as the precursor of flavin adenine dinucleotide (FAD) and flavin mononucleotide (FMN). These flavocoenzymes are essential for a wide range of oxidation–reduction reactions involved in energy production, biosynthetic metabolism, detoxification, and antioxidant defense mechanisms.^[1]^ Clinical manifestations of riboflavin deficiency are uncommon but classically include cheilosis, angular stomatitis, seborrhoeic dermatitis, and other mucocutaneous changes. The recommended daily intake varies from 0.4 mg in infants to 1.8 mg in young male adults, with higher requirements during pregnancy and lactation.^[1]^

Optimal activity of glutathione reductase depends on adequate availability of its flavin cofactor FAD. Impaired glutathione-reductase function may decrease cellular antioxidant capacity, a mechanism previously demonstrated in patients with rheumatoid arthritis (RA).^[2]^ More recent studies have also shown that individuals with systemic lupus erythematosus (SLE) frequently exhibit reduced riboflavin intake or biochemical evidence of flavin insufficiency, correlating with oxidative stress markers and subclinical atherosclerosis, suggesting a potential role for riboflavin-related pathways in SLE pathophysiology.^[3,4]^

In this regard, a study reported low riboflavin levels in approximately 33% of RA patients; notably, these patients exhibited higher pain scores, swollen joint counts, and increased C-reactive protein and erythrocyte sedimentation rate values compared with those with normal riboflavin status.^[2]^ In experimental models, riboflavin supplementation showed an inhibitory effect on spontaneous gonarthrosis development in mice.^[5]^

To further clarify this potential relationship, we conducted an extensive literature search in PubMed, SciELO, and LILACS databases (1965–May 2024) without language restriction. Interestingly, no clinical or experimental study directly assessing riboflavin supplementation in rheumatic diseases was identified.

Given this gap, preliminary research strategies should include controlled trials targeting rheumatoid arthritis and systemic lupus erythematosus, using riboflavin doses between 5–20 mg/day, evaluating disease activity scores (*e.g*., DAS28, SLEDAI), inflammatory biomarkers (CRP, ESR), oxidative-stress markers (GSH/GSSG ratio, MDA), and patient-reported outcomes such as pain and fatigue.

In conclusion, despite plausible biochemical and experimental evidence linking riboflavin to oxidative homeostasis, there is a complete absence of clinical trials evaluating its therapeutic potential in rheumatology. Controlled studies in rheumatoid arthritis and other autoimmune conditions are warranted to determine whether riboflavin supplementation could modulate disease activity or oxidative stress biomarkers.
